# Large Sample Area and Size Are Needed for Forest Soil Seed Bank Studies to Ensure Low Discrepancy with Standing Vegetation

**DOI:** 10.1371/journal.pone.0105235

**Published:** 2014-08-20

**Authors:** You-xin Shen, Wei-li Liu, Yu-hui Li, Hui-lin Guan

**Affiliations:** 1 Key Laboratory of Tropical Forest Ecology, Xishuangbanna Tropical Botanical Garden, Chinese Academy of Sciences, Menglun, Yunnan, China; 2 Restoration Ecology Group, Xishuangbanna Tropical Botanical Garden, Chinese Academy of Sciences, Kunming, China; 3 Kunming Metallurgical Research Institute, Kunming, China; 4 Yunnan Normal University, Kunming, China; University of Nevada, Reno, United States of America

## Abstract

A large number of small-sized samples invariably shows that woody species are absent from forest soil seed banks, leading to a large discrepancy with the seedling bank on the forest floor. We ask: 1) Does this conventional sampling strategy limit the detection of seeds of woody species? 2) Are large sample areas and sample sizes needed for higher recovery of seeds of woody species? We collected 100 samples that were 10 cm (length) ×10 cm (width) ×10 cm (depth), referred to as larger number of small-sized samples (LNSS) in a 1 ha forest plot, and placed them to germinate in a greenhouse, and collected 30 samples that were 1 m×1 m×10 cm, referred to as small number of large-sized samples (SNLS) and placed them (10 each) in a nearby secondary forest, shrub land and grass land. Only 15.7% of woody plant species of the forest stand were detected by the 100 LNSS, contrasting with 22.9%, 37.3% and 20.5% woody plant species being detected by SNLS in the secondary forest, shrub land and grassland, respectively. The increased number of species vs. sampled areas confirmed power-law relationships for forest stand, the LNSS and SNLS at all three recipient sites. Our results, although based on one forest, indicate that conventional LNSS did not yield a high percentage of detection for woody species, but SNLS strategy yielded a higher percentage of detection for woody species in the seed bank if samples were exposed to a better field germination environment. A 4 m^2^ minimum sample area derived from power equations is larger than the sampled area in most studies in the literature. Increased sample size also is needed to obtain an increased sample area if the number of samples is to remain relatively low.

## Introduction

The presence of viable seeds in the soil has been extensively documented [1]–[3], and seeds in the soil may include those dispersed directly from existing plants at the site, persisting from previous vegetation, and arriving via secondary dispersal from other sites [4]–[6]. Ecologists, evolutionary biologists and restorationists are interested in the composition and density of seeds in the soil seed bank and thus many studies have been conducted [2], [5]. However, the lack of consistent guidelines for sampling seed banks remains as an obstacle in making it possible to compare the results of seed bank studies, especially of forests.

In the great majority of studies, soil samples are taken by excavating soil cores or quadrats of varying depths, spreading the soil in trays under conditions suitable for germination, and identifying and counting the seedlings to calculate soil seed bank density and richness [4], [5], [7]. To save space and accelerate germination, soil samples can be sieved to reduce soil volume and then seeds are allowed to germinate [8], [9]. Seeds have clustered spatial distributions [10], which produce large sampling variances and may lead to imprecise abundance estimates. The precision (narrow confidence interval) of seed-number estimates may be improved by taking a large number of small samples (LNSS) [11]. This LNSS sampling strategy is successful in grasslands and agricultural lands for estimation of both seed density and diversity [11], [12]. However, in most forests, the LNSS method does not adequately detect seeds of woody species. Seeds of predominantly climax (woody) species in extant tropical forests often are absent from soil seed banks, but those of weedy and short-lived pioneer species can be abundant [13]. Clark *et al.* [14] reviewed the forest soil seed bank literature (90 studies) from 1969 to 1998 and found seeds of woody plants to be very rare, while those of herbaceous taxa were common. Hopfensperger [15] reviewed 108 articles published between 1945 and 2006 on the similarity between above and belowground species composition and found lower similarities in forests than in grasslands and in wetlands. Another comprehensive review from Europe also showed low similarities between forest-soil seed banks and standing vegetation [16]. However, on the forest floor, many species produce seedlings under a range of environmental conditions. This large discrepancy between lack of seeds in the soil seed bank studies and presence of seedlings limits valid interpretations of the soil seed bank and forest stand relationships, and it could lead to misjudgment of restoration potentials in degraded and disturbed forest sites.

Difficulties in detecting seeds of woody species in soil samples from forests may be attributed to spatially structured deposition patterns [17], short-lived seeds [16], ephemeral soil seed banks [15], and predation [18]. However, sampling methods, which could be part of the problem, are rarely discussed. Thus we need to consider minimum sample areas, the smallest area within which species are adequately represented, and the sample size, which varies widely among areas and research interests [14].

Appropriate sampled soil areas or volumes can be determined in a manner analogous to species-area curves for aboveground plants, and many such studies are found in the literature [19], [20]. Forcella [12] found that the combined soil surface areas of replicates should be about 1000 cm^2^ to ascertain the number of species with seed reserves in pasture soils in Australia. However, for forest soil seed banks, the species-area relationships suggest that a small sample size and soil surface areas might not yield a high detection percentage of woody species. For example, the largest number of species detected was only 34 in the accumulated species area curve for soil seed bank samples collected in a secondary tropical rain forest in Costa Rica [21]. These authors collected 121 soil samples (4.7 cm in diameter) at the intersection points of a 10×10 m grid in a 1 ha plot with a total area of 893 cm^2^. Although they did not separate woody species from herbaceous species, a low detection percentage of woody species could be inferred since this number of species was far lower than the 135.5±6.5/0.5 ha of understory species (not including canopy species) in the site [22]. Thus, we hypothesized that larger soil sample sizes and sampled areas will allow for more accurate determination of the presence and abundance of seeds of woody species in forest soils.

In our study, a large number of small soil sample (LNSS) and a small number of large sample (SNLS) were taken from a diverse karst forest in Yunnan Province of southern China, and the germination of woody species was determined. Our specific objectives were to: 1) Determine the effectiveness of the LNSS vs. the SNLS strategy in detecting woody-plant seeds in forest-soil seed banks; 2) Analyze the relationship between the number of woody species and sample area; 3) Suggest an appropriate sampling area and size for forest soil seed-bank studies.

## Material and Methods

### Study site

This study was conducted at Shilin Stone Forest Geographical Park (24°38′–24°58′N, 103°11′–103°29′E), Yunnan Province, southwest China. This area is 1950 m a.s.l., and it has a mean annual precipitation of 967.9 mm, 80% of which falls between May and October. Mean annual temperature is 16.2 °C, mean monthly maximum temperature is 20.7°C (July), and mean monthly minimum temperature is 8.2°C (January) [23]. The growing season coincides with the rainy season and with moderate temperature periods from late spring into summer [24]. The karst landscape consists of rock gaps, rock ditches, small rock caves and rock slots, with about 30% of the land surface being covered by soil. The soil is shallow and patchily distributed on or between these various rock surfaces. The primary forest on this karst land is an evergreen broadleaved forest mixed with few deciduous species [25], [26]. Most tree species shed their seeds in autumn (September to November) [27]. All necessary permits were obtained for field survey and soil replacement experiment from Shilin Stone Forest Management Bureau, Shilin County. No endangered species was involved.

**Figure 1:**
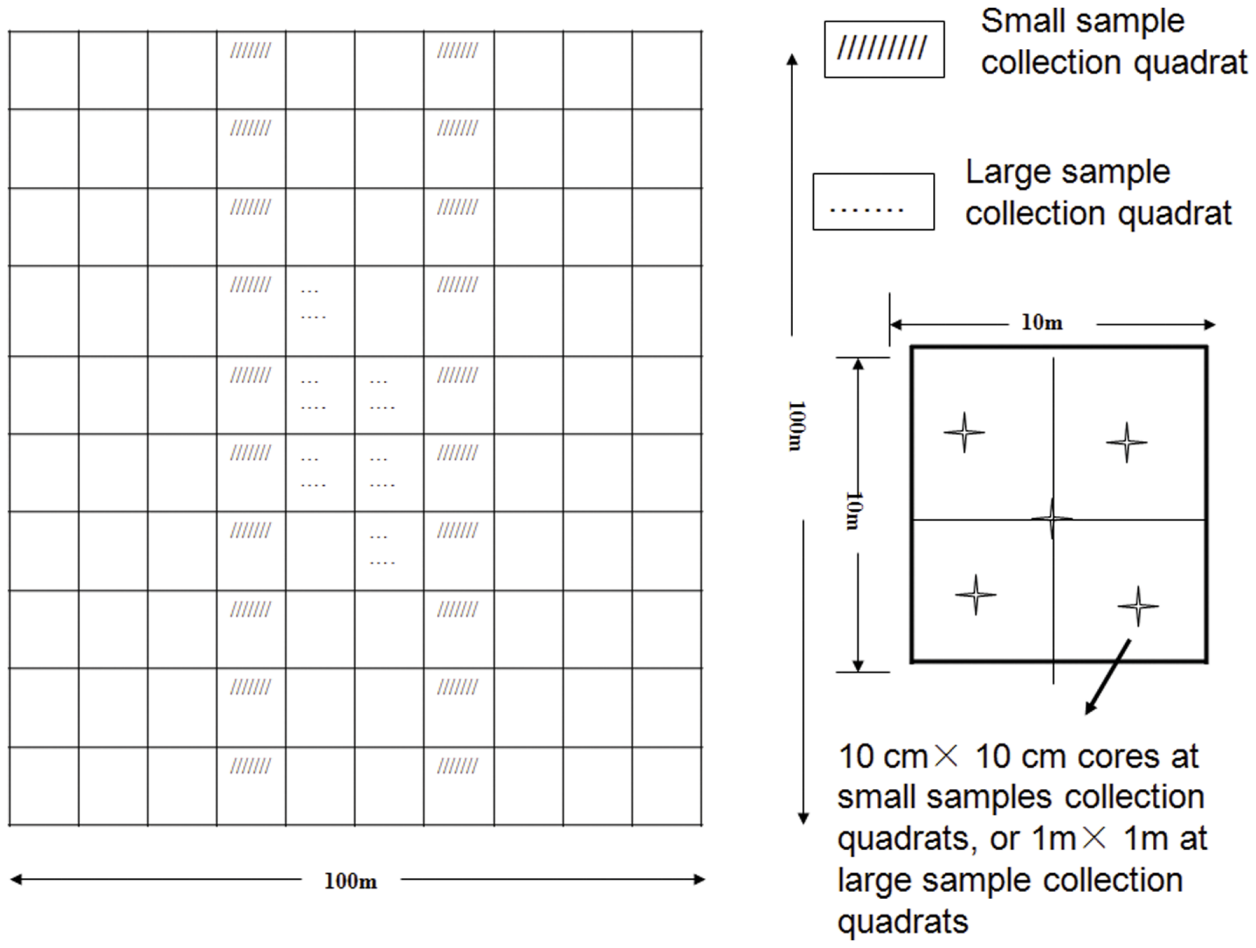
Illustration of sample designs for the vegetation survey and two types of soil sample collection for the soil seed bank study. A 100 m×100 m plot was divided into 10 m grids for the vegetation survey. Twenty quadrats of two vertical transects were selected for collection of a large number of small samples (LNSS) 100 cm×100 cm (slanted lines), and 6 quadrats were selected for collection a small number of large samples (SNLS) 1 m×1 m (dots).

A 100 m×100 m plot was established on a hill slope and divided into a 10 m grid (Fig. 1). In each of the quadrats (10 m×10 m), tree individuals with the main shoot ≥3 cm DBH (diameter at breast height) were identified and recorded. At the center of each quadrat, a 2 m×2 m small quadrat was established, and all woody individuals >30 cm height were identified and recorded.

### Soil sample collection methods

Two types of soil sample were collected to determine forest soil seed bank: 1) Large number of small samples (LNSS); 2) Small number of large samples (SNLS).

Two 100 m (length) ×10 m (width) vertical transects spaced 20 m apart along the slopes of the vegetation survey plot were selected. The transect was three grids wide (30 m) from one edge of the vegetation plot. All 20 quadrats in the two transects were chosen for LNSS. Six quadrats (10 m×10 m) at the most central part of the plot survey were selected for SNLS (Fig. 1). We further divided the sample quadrats into 5 m×5 m sub-quadrats. Soil samples 10 cm (length) ×10 cm (width) ×10 cm (depth) were collected at the center of the 20 LNSS sample quadrats and at the center of each sub-quadrat, thus 5 small sized soil samples were taken from each LNSS quadrat and 100 small samples were taken in total. Following the same arrangement, five 1 m (length) ×1 m (width) ×10 cm (depth) large sized soil samples were taken from each of SNLS quadrat and 30 larger-sized samples were taken in total.

All soil samples were collected at the end of November, when most of woody plants had shed their seeds. Those samples may contain both transient and persistent soil seeds [27]. Since the soil was patchily distributed, LNSS were taken nearest the marked sampling point to avoid collecting stones. For SNLS, we collected extra soil to compensate for soil-area loses if the 1 m×1 m sample sites were partially occupied by rock outcrops.

### Seed germination of LNSS

All LNSS samples were transported to the Xishuangbanna Tropical Botanical Garden, Kunming (80 km away from the Park, 1892 m a.s.l.) after collection. Each LNSS was washed through a 4 mm mesh sieve to eliminate coarse materials, and then through a 0.21 mm mesh sieve to eliminate fine materials [8]. Seeds larger than the 4 mm mesh were carefully collected and returned to the residues. The concentrated residue, which contained all seeds from the sample, was spread out evenly onto a 3 cm layer of perlite in plastic seed trays (approx. 18 cm×10 cm×10 cm deep). Trays were placed in a nonheated greenhouse in the Xishuangbanna Tropical Botanical Garden, Kunming, and seedling emergence was monitored. The sides of the greenhouse were covered by a double layer of fine nylon mesh cloth and the roof with a sheet of transparent polyethylene. Other trays containing sterilized soil were placed randomly within the greenhouse to monitor airborne seed contamination. All trays were monitored and watered, usually twice a day. Seedlings of woody species were counted and discarded as soon as they could be identified, or were transplanted into 15-cm-diameter pots filled with fertile soil and grown until species identification was possible. Soil in the trays was stirred two to three times during the germination monitoring period, normally after a large flush of germination. Recording was ceased at the end of the following November, 1 year after soil sample collections.

### Soil replacement and germination monitoring for SNLS

All the soil from the 30 SNLS was hand mixed, weighed and divided evenly into 30 parts (referred to as soil sources) to homogenize replicates.

Similar karst slopes covered with secondary forest, shrub and grass were chosen as recipient sites. There was no forest similar to that of the soil collection site in the areas surrounding the three recipient sites. Ten cm deep 1 m×1 m plots were prepared by removing all plants, soils and stones from each of the three sites. The 30 SNLS soil mixtures were transferred and placed on each of the 30 plots in all three recipient sites. A thin layer of rice straw was placed on the surface of each plot to limit moisture loss. The straw was removed between April 6–10, before the beginning of the rainy season (May). There were 1870–4800 seeds m^−2^ year round in primary forest in this study area [27], most of which were herbaceous. Counting those herbaceous seedlings in the 30 m^2^ field plots was highly labor intensive. Further, preventing local herbaceous seed entry to the plot was impossible as many herbaceous seeds were small in size and dispersed through the year. Thus, we excluded herb-seedlings counting and discarded all of them. Seedlings of woody species were identified and counted at half- month intervals from June to September 2009, and then at 1-month intervals until December.

The number of live and dead seedlings of each species in each plot was counted at each observation time (t). After recording, dead seedlings were removed to prevent recounting. We did not remove live seedlings in any plots since they might contribute to the restoration of degraded sites. We denote TSt(x) as the total number of seeds germinated of species x up to time t, NSt(x) as the total seedlings of species x at time t, NAt(x) as live seedlings of species x at time t, and NDt(x) as dead seedlings of species x at time t, so the total number of seedlings of a species at a plot at time t was:
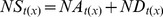



At counting time t+1, the number of seedlings of species x included the newly emerged ones, the live ones and dead ones of time t. The total number of seeds germinated at time t+1 was:


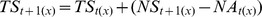
 if 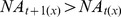 (if new seedlings emerged)

or


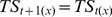
 if 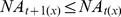 (if no new seedlings emerged).

Thus, for species x in plot i, the total number of seeds germinated was the TS(x) at the end of December. The total number of seeds germinated in a plot was the sum of seeds germinated for each species.

### Numerical analyses

The differences between total seed number and total species number were tested by one-way ANOVA among the three recipient sites for SNLS. Where main effects were significant (P<0.05), Duncan's multiple range tests were used to compare differences between means of seed germination between recipient sites.

We defined infrequent species as those with only one individual in the vegetation plot, or only one germinated seed in LNSS or SNLS.

We transferred each set of soil-replacement results and soil seed bank data (Appendix S1) to S (species)-by-H (quadrat) incidence matrices (presence-absence). Sample order was randomized 100 times to calculate a mean species accumulation curve within each plant survey plot, LNSS, and each of the recipient site for SNLS [28] in “EstimateS 9.10” [29]. Two-parameter power equations [30] often are used for species-area relationships [20], [31]. The mean predicted species accumulations (S) were fitted to power equations for the area resampled (A) (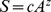) for vegetation, LNSS and each of the recipient site for SNLS.

## Results

Eighty-three woody species from 75 genera in 55 families were identified in the 1 ha vegetation plot (Appendix S1). Twenty-four species had only one individual in the vegetation plot. Seeds of 32 species from 30 genera in 19 families germinated from the three SNLS recipient sites (Appendix S1). The number of species identified at the secondary forest site, shrub site and grass site was 22.9%, 37.3% and 20.5%, respectively, of the total number of woody species in the 1 ha vegetation plot. However, only 13 species from 11 genera of 9 families were identified from LNSS in the greenhouse, which only accounted for 15.7% of woody species in the forest stand (Table 1). Seeds of the most abundant five tree species, *Cyclobalanopsis glaucoides, Olea yunnanensis, Pistacia weinmannifolia, Neolitsea homilantha, Carpinus mobeigiana*, which had >100 individuals in the 1 ha plot, were found in the SNLS (Appendix S1). However, only seeds of two of these species, *N. homilantha* and *C. mobeigiana*, were found in LNSS plots (Appendix S1). Generally, the chances of finding seedlings of abundant trees, shrubs and woody lianas were higher in SNLS than in LNSS (Appendix S1). Many species were infrequently (only germinated in 1 sample) encountered in both LNSS and SNLS plots (Table 1). Two *Smilax* species were present in the forest stand. However, the *Smilax* seedlings could not be identified at the species level. Eight species present in the soil seed bank were absent from the vegetation.

**Table 1:** Comparisons of species composition in the forest survey and in the soil seed bank using two collection strategies: large number of small samples (LNSS) and small number of large samples (SNLS) to replace soils of secondary forest, shrub and grass land.

	Plant survey	SNLS-Secondary forest	SNLS- Shrub	SNLS-Grass	LNSS
Sample size	10 m×10 m	1 m×1 m	1 m×1 m	1 m×1 m	**10 cm×10 cm**
No. of samples	100	10	10	10	**100**
No. of species per sample (mean ±SD)*	13.2 ±3.04	7.2±1.29^A^	10.8±1.54^B^	5.5±1.14^A^	**0.2±0.24**
No. of seeds/ m^−2^ *		33.4±4.6^B^	40.5±8.6^B^	15.9±2.4^A^	**355±62.9**
Total no. of species	83	19	31	17	**13**
Infrequent species	24	1	4	1	**7**
Similarity**	1	0.313	0.393	0.202	**0.226**
**Estimated richness**	**85**	**19**	**31**	**17**	**13**

*Different uppercase letters indicate the significant difference between two SNLS sites.

** = 2c/(a+b), where c is the number of common species, a is the number of species in the vegetation and b is the number of species in the soil seed bank.

On average, each 10 m×10 m forest stand plot contained 13.2±0.3 (mean ±SE) species, each 1 m×1 m SNLS geminated 5.5±1.14 (grassland) to 10.8±1.54 (shrub land) woody species via soil replacement, and each 10 cm×10 cm LNSS germinated only 0.2±0.24 woody species (Table 1). The number of seeds germinated from 1 m^2^ soil in LNSS in greenhouse is far larger than any one of the SNLS in the field.

The number of species (F = 6.49, P = 0.005) and the number of seeds (F = 4.76, P = 0.017) that germinated in the three recipient sites differed significantly. Both the highest number of species and number of seeds were found at the shrub site (Table 1).

**Figure 2:**
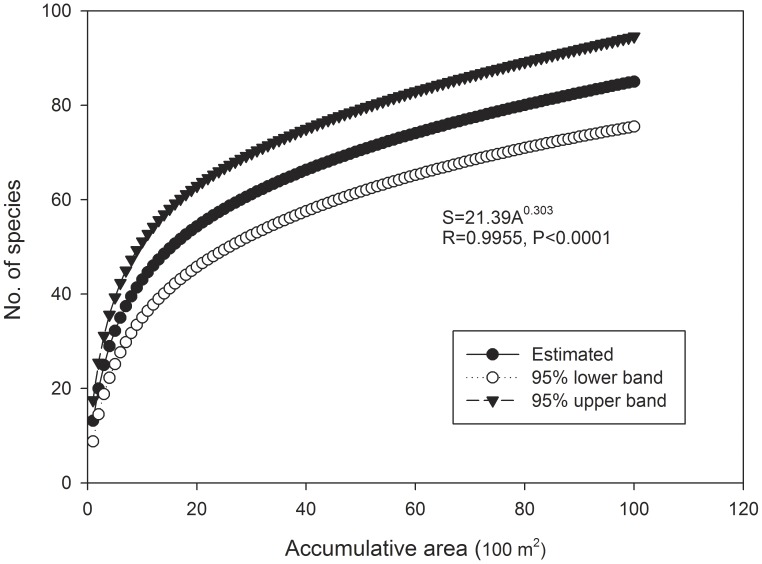
Mean woody species accumulation curve and 95% confidence intervals for karst forest in SW China. Samples were pooled from 100 quadrats of a 100 m×100 m vegetation survey and then randomized 100 times in “EstimateS 9.10” (Colwell 2013).

**Figure 3:**
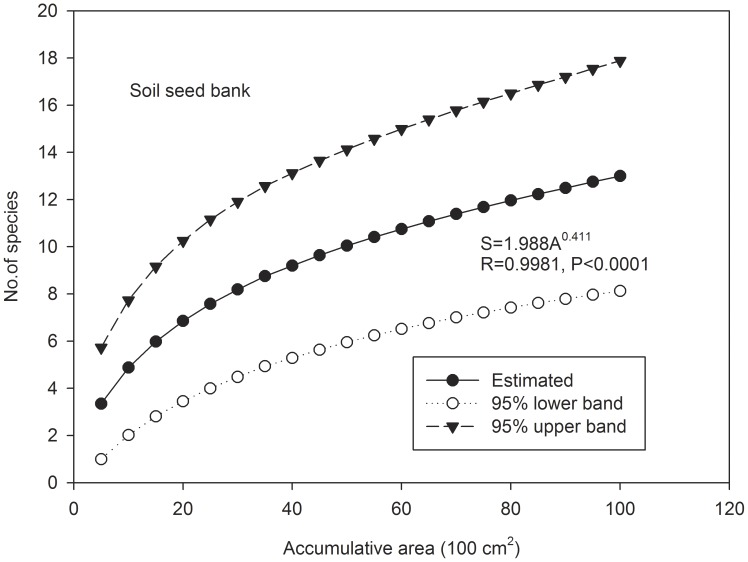
Mean woody species accumulation curve of seeds and its 95% confidence intervals in soil seed bank of a karst forest in SW China. Samples were pooled from 100 quadrats of the soil seed bank by collecting 10 cm×10 cm×10 cm small soil samples and then randomized 100 times in “EstimateS 9.10” (Colwell 2013).

**Figure 4:**
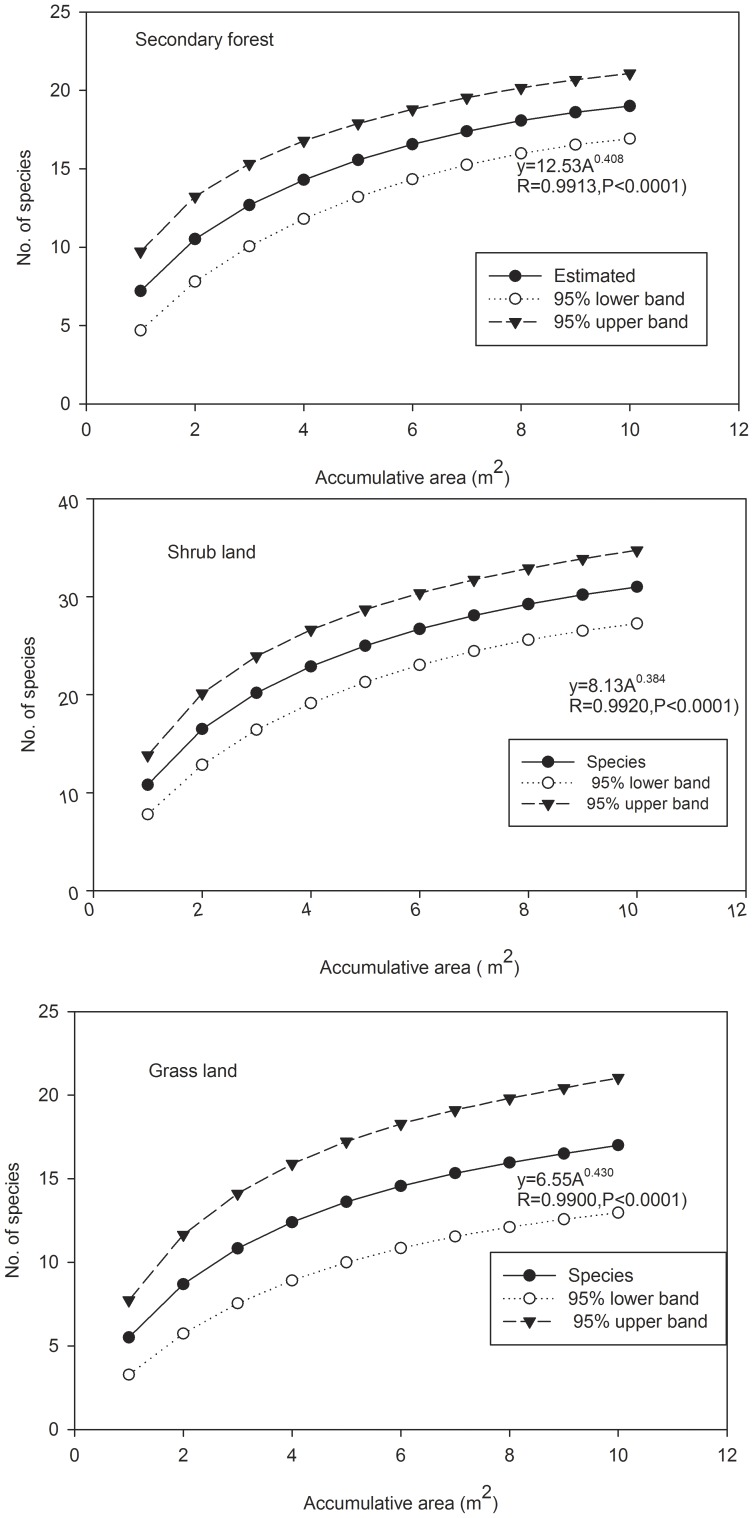
Mean woody species accumulation curve of seeds and 95% confidence intervals in soil seed bank of a karst forest in SW China. Samples were pooled from 10 quadrats of the soil seed bank by collecting ×1 m larger soil samples to replace soil plots of secondary forest, shrub and grass land and then randomized 100 times in “EstimateS 9.10” (Colwell 2013).

The number of species initially increases steeply with the sample areas pooled for vegetation plots, SNLS and LNSS, but then approaches an asymptote, in accordance with the power-law equation (Fig. 2, 3, 4). The simulated richness was 102.4% of the number of species observed in the 1 ha vegetation survey plot, and it was equal to the number of species identified in the three recipient sites of SNLS method and of species identified in the SNLS method.

## Discussion

Even with 100 small-sized samples only 13 woody species were found by the LNSS method. This number is far lower than the number of woody species identified in the vegetation survey (83 species, Table 1), even excluding infrequent species (24 species that have only one individual each in vegetation plot). However, this result coincides with most other forest soil seed-bank studies, such as in North America [14], Europe [16], tropical wet forests [21], and central African rainforests [13]. On the other hand, 31, 19, and 17 species were identified in a total of 10 SNLS (1 m×1 m size) at the shrub, the secondary forest and grass recipient sites; this was 33.7%, 21.7% and 18.1%, respectively, of the total number of woody species in the 100 m×100 m vegetative plot. In addition, 84.5%, 51.8% and 46.3% of the estimated number of species in the six quadrats, from which SNLS samples were collected (by the power-law equation from the plant survey (Fig. 2), where A = 6×100 m^2^, S = 36.7). The SNLS method captured seeds of all dominant tree species in the standing vegetation. This means that by collecting 10 samples 1 m×1 m in size and placing them in an environment conducive to germination, such as that of shrub site in this study, a woody-plant seed bank that more accurately describes the forest stand was detected.

As germination conditions for LNSS and SNLS samples were not identical, comparing them may present some limitations. The differences among the three recipient sites provide information about the importance of germination environments (Table 1). However, the germination environment of SNLS, even at the most favorable field germination site - the shrub recipient site, may not equal the environment for LNSS in the greenhouse. Thus, empirically, the number of species and seedlings germinated at the three recipient sites were not at the maximum. This means a lower estimation of detection percentages on woody species might be obtained from the three recipients. So, we can confidently say the 10 SNLS (1 m×1 m size) had a higher percentage of detection for woody species than the 100 SNSS (10 cm×10 cm size).

Sample area size (A) is the fundamental element to determine the number of species in soil seed bank studies. The species–area relationship has traditionally been approximated by a power law [20], and it is widely applied to describe the pattern of increasing numbers of plant species with study-area size [20], [28], [29], as well as in soil seed bank studies [21]. The species-area curves in our study all follow power law pattern (S = cA^z^) in stand forest, LNSS and SNLS methods (Figs. 2, 3, 4), by which the number of species against sampling area initially increases steeply but then approaches an asymptote.

The z-values in the power-law equation describes the rate of accumulation of species and the c value describes the interception with the increase of area in the logarithmic space 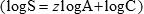. These two parameters are affected by the sample size/total area, number of species per sample, etc. [19]. In our study, lower z-value variations were found among equations for the stand forest (0.303, Fig. 2), LNSS (0.411, Fig. 3), and the three SNLS equations at different recipient sites (0.408, 0.364, 0.430, Fig. 4); these z values were also within the range (0.064 to 1. 312) of those found in an inland species-area data analysis [19]. However, the c value varied greatly among the three equations, possibly affected by sample sizes since the c value was (i) lowest in the LNSS method (1.988, Fig. 3), in which sample size was the smallest (100 cm^2^), (ii) highest in the plant survey (21.39, Fig 2) in which sample size was the largest (100 m^2^), and (iii) an intermediate value in the SNLS method (12.53, 8.13, 6.55, Fig. 4) in which sample size was intermediate (1 m^2^). However, unlike the stand forest, the c value for the soil seed bank also was affected by germination environments, as it varied among the three recipient sites.

Following the methods used in vegetation surveys, a minimum area can be determined from the power-law equations for forest soil seed-bank studies. The 10% ratio, which is “the point along the species determined / sample area size curve at which a 10% increase of the total sampling area yields only 10% more species of the total number recorded,” is one criterion to determine suitable minimum areas [32], [33]. Using the constructed equations to calculate the number of species at a stimulated sample area, we found that before the LNSS reaches 2000 cm^2^, or SNLS reaches 4 m^2^, the increase percentages of species determined will be greater than 10%. Correspondingly, predicted S (number of species) was 7 in LNSS, 23 in SNLS at shrub recipient site, 14 in SNLS at secondary forest recipient site, and 12 in SNLS at grass recipient site. This means that the 2000 cm^2^ minimum area under LNSS would reach saturation with 8.2% of the species of the 1 ha plot and 27.1%, 16.5% and 14.1% in the 4 m^2^ minimum area under SNLS by germination at shrub, secondary forest and grass recipient sites, respectively. Since the SNLS was collected from only six quadrats, the rates of saturation also could be 62.7%, 38.1% and 32.7% at shrub, secondary forest and grass recipient sites (by the power-law equation of plant survey (Fig. 2), when A = 6×100 m^2^, S = 36.7). Thus, we can take 4 m^2^ as the minimum area for forest soil seed bank studies in our study area to fulfill the 10% increase ratio and also to yield a higher saturation ratio (2/3 at the most favorable recipient site) of a stand forest plot. This minimum area is a far larger area than the recommended minimum areas in agricultural land [12]. Unfortunately, most forest soil seed bank studies do not use as large a minimum area as in our study. For instance, Clark *et al.* [14] reviewed 90 forest soil seed-bank studies and found most A<0.2 m^2^, which is far smaller than the minimum area in this study. Thus, we recommend a large sample area to ensure a high detection percentage on woody species.

In the power-law equation, the estimated number of species can be expressed as: 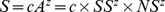. Theoretically, an increase of either SS (sample size) or NS (number of sample) can cause S (number of species) to increase. A large number of small samples (LNSS) presents logistical problems, particularly if seeds are to be counted by germination in a greenhouse. Further, an increased number of samples would be limited since each sample must be bagged, potted, counted, and cared for separately, and this can be very time consuming. For this reason, presumably, many researchers have chosen to use relatively large sample-unit sizes. Resolution could be found by taking smaller samples from large quadrats and pooling them together [11]. Even with this arrangement, greenhouse germination and seedling care usually take a long time, especially for forest soils. A smaller sample size also may reduce the soil depth and expose large seeds, potentially reducing seed germination and seedling survival. On the other hand, collection of a small number of larger samples may damage the forest floor, and uncontrolled field environments would bring larger variation in results (Table 1). Thus, we need to maintain a balance between sample size and number of samples for empirical and practical reasons. Theoretically, if we take samples larger than 10 cm×10 cm, i.e. 20 cm×20 cm, and keep the same collection method as the 10 cm×10 cm, we can get a minimum area  = 4 m^2^, and S = 23.3 following the existing LNSS power-law equation (Fig. 3). This is 28.1% of the total plant species within the 1 ha vegetation plot.

Several studies propose a small sample size but large number of samples to account for clustered spatial distribution of seeds [7], [11]. We might have anticipated that the LNSS sampling method would yield higher S than the SNLS method when the same A (sample area) value was considered. The simulation following the power-law equation of LNSS (Fig. 3) and of SNLS for the shrub site (Fig. 4) did not show a sharp difference between these two methods (i.e, when A = 2 m^2^, S = 17.5 for LNSS and S = 16.6 for SNLS shrub site, when A = 3 m^2^, S = 20.7 for LNSS and S = 19.6 for SNLS shrub site). Thus, the sample size in our forest soil seed bank study did not yield the anticipated effect. Probably, mixing SNLS samples before transplantation into recipient plots mitigated this clustering effect, and clustered spatial distributions could be mitigated by increased number of samples and increased sample sizes. On the other hand, a reasonable number of samples could provide enough replication to control for statistical variations. Thus, a minimum number of samples should be maintained.

Our study confirms the shortage of conventional large number of small sized samples (LNSS) in detecting seeds of woody species. This may in part explain low woody-plant seed detection [34] and low similarities between forest stands and soil seed banks [15], since most studies in the literature had a smaller sampling area [14], [35] than ours (1 m^2^ in total for the 100 LNSS). Consequently, this high discrepancy has limited applications of soil seed-bank results in forest ecology and restoration studies [13], [16]. Our study also demonstrates that seeds of species dominant in the forest stand could be detected by increased total sample areas and sizes such as SNLS. This would provide more information to explain regeneration and recruitment dynamics of woody populations, communities, and also to assess the restoration capability of degraded forest sites both. Thus, a large sample area and size are needed for forest soil seed bank studies to detect woody species to ensure low discrepancy with stand forest. In practice, larger sample areas and sizes may be difficult to achieve and might also damage forest soil floors. We can compensate for the area increase by reducing the sample depth in soil volume, for instance, we can reduce the depth to 3 to 5 cm in most forest soils [36], [37] since woody seeds are not efficiently transferred to deep soil layers [4], [7]. Reducing the amount of soil could also reduce the burden of transporting soil samples to the greenhouse and facilitate germination in greenhouse, thus help mitigate germination variation in noncontrolled natural environments, such as our three recipient sites.

Due to our study's design, the field germination environment of a small number of large sized samples (SNLS) might be less favorable as that of LNSS in the greenhouse, together with variation in the field germination environment at the three recipient sites, we might not get the maximum detection percentage of woody species to construct species-area curves for SNLS sample strategies. On the other hand, we did not consider the fine-scale (i.e. within 1 m×1 m SNLS in our study) spatial seed bank structure, which could be used to design lumped soil cores in reducing soil collection amount of the sample [10]. These would limit the precision in estimating the minimal sample area and sample size. Thus, 4 m^2^ as the minimum area fulfilling the 10% increase ratio might not be precise for forest soil seed bank studies in our study area and similar areas. However, 4 m^2^ might be the first estimation of minimum area for forest soil seed bank studies in a particular forest. Thus, it should be further tested at other study areas.

## Supporting Information

Appendix S1 **The number of individuals in vegetation stand and number of seedlings germinated from 10 small number of large samples (SNLS) at secondary forest, shrub and grass recipient sites and from 100 large number of small samples (LNSS).**
(DOCX)
