# Supplementary material for: Large Sample Area and Size Are Needed for Forest Soil Seed Bank Studies to Ensure Low Discrepancy with Standing Vegetation
Source: PLoS One. 2014 Aug 20;9(8):e105235. doi: 10.1371/journal.pone.0105235 (PMC4139333; doi:10.1371/journal.pone.0105235)
Supplement: Appendix S1 — The number of individuals in vegetation stand and number of seedlings germinated from 10 small number of large samples (SNLS) at secondary forest, shrub and grass recipient sites and from 100 large number of small samples (LNSS). (DOCX) [file pone.0105235.s001.docx]

Appendix S1 The number of individuals in vegetation stand and number of seedlings germinated from 10 small number of large samples (SNLS) at secondary forest, shrub and grass recipient sites and from 100 large number of small samples (LNSS)

| **Name** | **Life form*** | **Plant survey**** | **SNLS-secondary forest** | **SNLS-shrub** | **SNLS-grass** | **LNSS** |
| --- | --- | --- | --- | --- | --- | --- |
| *Cyclobalanopsis glaucoides* Schottky | t | 944 | 40 | 23 | 2 |  |
| *Olea yunnanensis* Hand-Mazz | t | 521 | 1 | 4 |  |  |
| *Pistacia weinmannifolia* J.Poisoon ex Fr. | t | 461 | 58 | 35 | 6 |  |
| *Neolitsea homilantha* Allen | t | 403 | 52 | 30 |  | 1 |
| *Carpinus mobeigiana* Hand.-Mazz | t | 121 | 7 | 25 |  | 13 |
| *Rhamnus leptophylla* Schneid | t | 86 | 41 | 44 | 18 |  |
| *Toxicodendron griffithii*.(Hook.f.) O.ktze | t | 83 |  |  |  | 1 |
| *Ficus virens* Ait. | t | 61 |  |  |  | 14 |
| *Toxicodendron succedaneum* (L.) O.kuntze | t | 60 |  |  |  |  |
| *Ilex macrocarpa* Oliv. | t | 50 |  |  |  |  |
| *Pistacia chinensis* Bunge | t | 49 |  | 28 | 5 |  |
| *Machilus yunnanensis* Lecomie | t | 41 |  | 2 |  |  |
| *Albizia mollis* (Wall.)Boiv. | t | 34 | 5 | 12 | 31 | 42 |
| *Pittosporum brevicalyx* (Oliv.) | t | 33 |  |  |  |  |
| *Celtis bungeana* Bl. | t | 30 |  |  |  |  |
| *Lindera communis* Hemsl | t | 28 | 7 | 6 |  |  |
| *Distyliopsis laurifolia* (Hemsl.)Endress | t | 19 |  |  |  |  |
| *Prunus zippeliana* Miq. | t | 16 |  |  |  |  |
| *Pentapanax henryi* Harms | t | 15 |  |  |  |  |
| *Dichotomanthes tristaniaecarpa* kurz | t | 12 |  |  |  |  |
| *Rhamnella martini* (Levl) Schneid. | t | 12 | 23 | 8 | 9 |  |
| *Xylosma racemosum* (Sieb et Zucc. ) Miq. | t | 11 |  |  |  |  |
| *Ficus pandurata* Hance | t | 9 |  |  |  |  |
| *Lithocarpus confinis* Huang et Chang | t | 6 |  |  |  |  |
| *Photinia glomerata* Rehd. Et wils | t | 6 |  |  |  |  |
| *Crataegus scabrifolia* (Franch.)Rehd. | t | 5 |  |  |  |  |
| *Rhus chinensis* Mill | t | 5 |  | 1 |  |  |
| *Fortunella sagittifolia* Feng et Mao | t | 4 |  |  |  |  |
| *Morus australis* Poir. | t | 4 |  |  |  |  |
| Photinia sp. | t | 4 |  |  |  |  |
| *Pyrus pashia* Buch.-Ham.ex. D.Don | t | 4 |  |  |  |  |
| *Reevesia pubescens* Mast. | t | 4 |  |  |  |  |
| *Ulmus changii* Cheng var. Kunmingensis (cheng)cheng et L.K.Fu | t | 4 |  |  |  |  |
| *Ficus chartacea* Wall.ex King | t | 2 |  |  |  |  |
| *Morus mongalica* (Bur.)Schneid. | t | 2 |  |  |  | 1 |
| *Broussenetia papyrifera* (L.)L'herites exVent. | t | 1 |  |  |  |  |
| *Celtis tetrandra* Roxb. | t | 1 | 3 | 5 |  |  |
| *Cornus paucinervis* Hance | t | 1 |  |  |  |  |
| *Ehretia corylifolia* C.H. Wright | t | 1 |  |  |  | 1 |
| *Quercus variabilis* Bl | t | 1 |  |  |  |  |
| *Neocinnamomum delavayi* (Lecomte)H.Liou | t |  |  | 7 |  |  |
| *Myrsine semiserrata* wall. | s | 94 |  | 4 |  |  |
| *Myrsine africana* L. | s | 72 | 11 | 13 | 7 |  |
| *Gardneria multiflora* Makino | s | 20 |  |  |  |  |
| *Leptopus chinensis* (Bunge) Pojarkova | s | 19 |  |  |  |  |
| *Nothapodytes tomentosa* C. Y.Wu | s | 17 |  |  |  |  |
| *Sageretia theezans* Linn. | s | 17 |  |  |  |  |
| *Helwingia himalacia* Hook.f. et Thoms | s | 15 |  |  |  |  |
| *Pouzolzia sanguinea* (Bl.)Merr. | s | 10 |  | 12 |  |  |
| *Diospyrus mollifolia* Rehd. et wils. | s | 4 |  | 7 |  |  |
| *Schoepfia jasminodora* S.et Z. | s | 4 |  |  |  |  |
| *Osteomeles schwerinae* Schneid | s | 2 |  |  |  |  |
| *Barleria cristata* Linn. | s | 1 |  |  |  |  |
| *Buddleia officinalis* Maxim | s | 1 |  |  |  | 3 |
| *Debregeasia edulis* (Sieb.et Zucc.)Wedd. | s | 1 |  |  |  |  |
| *Elaeagnus lanceolata* Walb. | s | 1 |  |  |  |  |
| *Indigofera cinerascens* Franch. | s | 1 |  |  |  |  |
| *Leptodermis potaninii* Batain | s | 1 |  |  |  |  |
| *Murraya paniculata* (Linn.)Jack. | s | 1 |  |  |  |  |
| *Osyris wightiana* wall. | s | 1 |  | 2 | 1 |  |
| *Sarcococca ruscifolia* stapf | s | 1 |  |  |  |  |
| *Spiraea japonica* L.f | s | 1 |  |  |  | 3 |
| *Campylotropsis polyantha* (Franch.)A.k.Schindl. | s |  |  | 26 | 4 |  |
| *Rubus delavayi* Franch | s |  |  | 1 | 7 |  |
| *Rubus obcordatus* Nauyea | s |  |  | 1 |  |  |
| *Rubus parvifolius* L. | s |  |  |  |  | 237 |
| *Sophora davidii* (Fr.)Komarov ex Pavd. | s |  |  | 4 | 6 |  |
| *Jasminum humile* Linn. | l | 110 |  |  |  |  |
| *Zanthoxylum scandens* Bl.(=cuspidata) | l | 55 | 10 | 41 | 38 | 1 |
| *Smilax glabra* Roxb | l | 40 |  |  |  |  |
| *Milletia dielsiana* Harms | l | 31 | 3 | 2 |  |  |
| *Dalbergia mimosoides* Franch. | l | 30 | 23 |  |  |  |
| *Trachelospermum bodinieri* (Levl.)Woods.ex Rehd. | l | 20 | 26 | 5 | 2 |  |
| *Jasminum nudiflorum* Lindl. | l | 16 |  |  |  |  |
| *Celastrus angulatus* Maxim | l | 15 | 2 | 1 |  |  |
| *Lonicera japonica* Thunb. | l | 14 |  |  |  |  |
| *Jasminum polyanthum* Franch. | l | 11 |  |  |  |  |
| *Smilax microphylla* C.H.Wripht | l | 8 |  |  |  |  |
| *Jasminum officinale* Linn. | l | 6 |  |  |  |  |
| *Milletia reticulata* Benth. | l | 4 |  |  |  |  |
| *Smilax ferox* Wall. | l | 4 |  |  |  |  |
| *Zanthoxylum armatum* DC. | l | 4 |  |  |  | 15 |
| *Clematis fasciculiflora* Franch | l | 2 |  |  |  |  |
| *Cocculus orbiculatus* (L.) DC. | l | 2 |  |  |  |  |
| *Paederia scandens* (lour.) Merr. | l | 2 |  |  |  |  |
| *Schisandra sphenanthera* Reht.et Wils. | 7 | 2 |  |  |  |  |
| *Clematis ranunculoides* Franch | l | 1 |  |  |  |  |
| *Euonymus fortunei* Maxim. | l | 1 |  |  |  |  |
| *Lonicera koehneana* Rehd. | l | 1 |  |  |  |  |
| *Ficus ti-koua* Bur. | l |  |  |  | 5 | 23 |
| *Smilax* sp. | l |  | 16 | 49 | 13 |  |
| *Apodytes dimidiata* E.Meyer |  |  | 2 |  |  |  |
| **Total No. of species** |  | **83** | **19** | **31** | **17** | **13** |

* t: tree, s: shrub, l: woody liana; ** The total number of trees with the main shoot ≥3 cm DBH in all 10 m × 10 m quadrats, the total number of shrub and the number of liana in all 2 m × 2 m small quadrats
